# Age- and time-dependent increases in incident anti-glomerular basement membrane disease: a nationwide cohort study

**DOI:** 10.1093/ckj/sfad261

**Published:** 2023-10-16

**Authors:** Karl Emil Nelveg-Kristensen, Bo Madsen, Mark McClure, Nanna Bruun, Cecilie Lyngsø, Hans Dieperink, Jon Waarst Gregersen, Elizabeth Krarup, Per Ivarsen, Christian Torp-Pedersen, Martin Egfjord, Wladimir Szpirt, Nicholas Carlson, for the Danish Vasculitis Association (DANVAS)

**Affiliations:** Department of Nephrology, Rigshospitalet, Copenhagen University Hospital, Copenhagen, Denmark; Department of Nephrology, SLE and Vasculitis Clinic, Aalborg University Hospital, Aalborg, Denmark; Vasculitis and Lupus Clinic, Addenbrooke's Hospital, Cambridge University Hospitals, Cambridge, UK; Department of Nephrology, Rigshospitalet, Copenhagen University Hospital, Copenhagen, Denmark; Department of Nephrology, Zealand University Hospital, Roskilde, Denmark; Department of Nephrology, Odense University Hospital, Odense, Denmark; Department of Nephrology, SLE and Vasculitis Clinic, Aalborg University Hospital, Aalborg, Denmark; Department of Nephrology, Herlev Hospital, Copenhagen University Hospital, Copenhagen, Denmark; Department of Nephrology, Aarhus University Hospital, Aarhus, Denmark; Department of Clinical Medicine, Aarhus University, Aarhus, Denmark; Department of Cardiology, Nordsjaellands Hospital, Hillerød, Denmark; Department of Public Health, University of Copenhagen, Copenhagen, Denmark; Department of Nephrology, Rigshospitalet, Copenhagen University Hospital, Copenhagen, Denmark; Department of Nephrology, Rigshospitalet, Copenhagen University Hospital, Copenhagen, Denmark; Department of Nephrology, Rigshospitalet, Copenhagen University Hospital, Copenhagen, Denmark

**Keywords:** age, analysis, epidemiology, gender, prognosis, survival

## Abstract

**Background:**

Epidemiologic assessments of anti-glomerular basement membrane (GBM) disease have been challenging due to its rare occurrence. We examined changes in the incidence and outcomes from 1998 to 2018 using nationwide healthcare registries.

**Methods:**

All patients with incident anti-GBM disease were identified using the International Classification of Diseases, 10th Revision code DM31.0A. Controls were matched 4:1 on birthyear and sex using exposure density sampling. Log link regression adjusted for time, age and sex was applied to model survival.

**Results:**

We identified 97 patients with incident anti-GBM disease, corresponding to an incidence of 0.91 cases/million/year [standard deviation (SD) 0.6]. The incidence increased over time [1998–2004: 0.50 (SD 0.2), 2005–2011: 0.80 (SD 0.4), 2012–2018: 1.4 (SD 0.5); *P* = .02] and with age [0.76 (SD 0.4), 1.5 (SD 1.04) and 4.9 (SD 2.6) for patients <45, 45–75 and >75 years]. The median age was 56 years (interquartile range 46) and 51.6% were female. Dialysis was required in 58.4%, 61.9% and 62.9% of patients at day 30, 180 and 360, respectively. The 1-year kidney survival probability was 0.38 (SD 0.05) and exhibited time-dependent changes [1998–2004: 0.47 (SD 0.13), 2005–2011: 0.16 (SD 0.07), 2012–2018: 0.46 (SD 0.07); *P* = .035]. The 5-year mortality was 26.8% and mortality remained stable over time (*P* = .228). The risk of death was greater than that of the matched background population {absolute risk ratio [ARR] 5.27 [confidence interval (CI) 2.45–11.3], *P* < .001}, however, it was comparable to that of patients with anti-neutrophil cytoplasmic antibody–associated vasculitis (AAV) requiring renal dialysis at presentation [ARR 0.82 (CI 0.48–1.41), *P* = .50].

**Conclusion:**

The incidence of anti-GBM disease increased over time, possibly related to temporal demographic changes. Mortality remained high and was comparable with an age- and sex-matched cohort of dialysis-dependent AAV patients.

KEY LEARNING POINTS **What was known:**

- Anti- glomerular basement membrane (GBM) disease is a rare small vessel vasculitis that affects glomerular and pulmonary capillaries, resulting in rapidly progressive glomerulonephritis and pulmonary haemorrhage.
- Anti-GBM disease is associated with a high risk of chronic renal failure and death.
- Efforts to accurately define the incidence and long-term outcomes in epidemiologic studies have been challenging due to the rarity of this disease.

**This study adds:**

- Nationwide data on 97 patients with anti-GBM disease from 1998 to 2018 was used.
- The incidence of anti-GBM disease increased over the test period, possibly related to better disease awareness, higher testing frequency and a demographic shift towards a larger population of older people.
- Mortality remained high and was comparable with an age- and sex-matched cohort of dialysis-dependent patients with neutrophil cytoplasmic antibody–associated vasculitis.

**Potential impact:**

- Better understanding of the association between temporal changes in anti-GBM disease incidence, anti-GBM testing frequency, age and double positivity of anti-neutrophil cytoplasmic antibody and anti-GBM serology.
- Emphasis on optimized methods for identifying patients at risk of contracting anti-GBM disease, with a specific focus on renal involvement and the need for dialysis at diagnosis, which is a marker of severe high-risk disease with poor chances of renal survival.
- Improved awareness of the age- and sex-dependent differences in clinical presentation and outcomes.

## INTRODUCTION

Anti-glomerular basement membrane (GBM) disease is a small vessel vasculitis affecting the kidneys and lungs. It is caused by the development of directly pathogenic autoantibodies targeting epitopes on the α3 chain of collagen type IV found in the glomerular and alveolar basement membrane [1, 2]. Anti-GBM disease is characterized by rapidly progressive glomerular disease (RPGN) and pulmonary haemorrhage, potentially leading to organ failure requiring renal replacement therapy (RRT) and invasive ventilation, with a subsequent high risk of chronic renal failure and death.

Epidemiological studies in anti-GBM disease are challenging, as it is a rare disease with an estimated annual incidence of 1–1.5 cases/million/year [1, 2] and large nationwide populations of unselected patients are not readily accessible. To date, few studies have assessed the incidence based on nationwide data and have instead relied on collaborations between selected centres within one country or between several different countries, which complicates classic epidemiologic assessments, and are prone to inaccurate calculations of time- and age-dependent alterations, as they rely on selected data. Here we examined the incidence and outcomes based on unselected data from the Danish nationwide healthcare registries in the period 1998–2018.

## MATERIALS AND METHODS

### Patients, case ascertainment and registries

Danish nationwide administrative registries, i.e. the National Patient Registry, the Danish Registry of Medicinal Product Statistics, the National Causes of Death Registry and the Central Person Registry as described previously [3], were used to extract data on all patients diagnosed with anti-GBM disease defined by the International Classification of Diseases, 10th Revision (ICD-10) code DM31.0A between 1998 and 2018. Information on anti-GBM testing was based on blood samples obtained from four of five administrative regions in Denmark during 2013–2018. To increase the sensitivity of the diagnostic codes, prescriptions of glucose-lowering drugs (A10) and loop diuretics (C03C) were used as proxies for diabetes and congestive heart failure [4]. Similarly, we defined hypertension as treatment with two or more antihypertensive drugs within a period of 3 months or any hospital admission with a hypertension diagnosis (ICD-8: 400–404; ICD-10: DI10-15) [5]. Baseline renal function was assessed by the need for dialysis [end-stage kidney disease (ESKD)] or the development of chronic kidney disease (CKD; defined by ICD-10 diagnostic codes DN02–08, DN11–12, DN18–19, DN26, DN158–159, DN162, DN164, DN168, DI120), and chronic dialysis was defined as the need of recurrent dialysis for >60 days. Advanced disease severity was defined as an initial hospital stay lasting >10 days or death within 10 days from admission.

We assessed the ICD-10 diagnostic code associated with anti-GBM disease (DM31.0A) in a pilot study of 19 patients from the northern region of Denmark who were diagnosed with anti-GBM disease in the Danish National Patient Registry during the years 2000–2019 and found a positive predictive value (PPV) of 100% [confidence interval (CI) 83–100]. All patients were identified by ICD-10 code and the anti-GBM disease diagnosis was manually cross-referenced in the patients’ local medical records. A diagnosis of anti-GBM disease was considered true if there was an elevated anti-GBM antibody in conjunction with biopsy-proven crescentic glomerulonephritis and/or alveolitis, with linear staining of immunoglobulin G (IgG) on immunofluorescence. The PPV was calculated as the proportion of diagnoses in the national patient registry confirmed by medical records review, with 95% CIs according to the Wilson score method [6].

### Statistics

The chi-squared test or Fisher’s exact test and Student's *t*-test or Mann–Whitney exact test were used to examine differences in categorical and continuous variables, respectively. One-way analysis of variance (ANOVA) was employed to test differences in means between groups. Local weighted scatterplot smoothing (LOESS) was applied to fit smooth curves through points in a scatterplot based on local weighted regression. The crude incidence of anti-GBM disease was expressed as cases/million/year. The cumulative incidence was computed based on the Aalen–Johansen estimator and assessed by Gray's non-parametric test, and mean follow-up time was calculated by use of reverse Kaplan–Meier. Unadjusted, time-specific Cox regression was used to model absolute survival probability at 1 year, expressed in percent. Absolute risk regression models, based on log link cumulative incidence regression [7], were used to model survival expressed as absolute risk ratios (ARRs). The AAR models were adjusted for year of inclusion, age and sex. In analyses of survival data, patients entered the model at the date of admission for the primary hospitalization when the anti-GBM diagnosis was initially confirmed and non-hospitalized patients diagnosed from outpatient clinics entered the model at the day of diagnosis. For patients with an unclear date of diagnosis, the first day of dialysis or first day of plasma exchange (PLEX) in association with that diagnosis was applied as the index. Patients were subsequently followed until a primary endpoint occurred (death), emigration, a maximum of 5 years or until 31 December 2018, whichever came first. A background population matched 4:1 by exposure density sampling on birth year and sex were included as controls in the assessment of mortality risk. Two-sided *P*-values ≤.05 were considered significant. Analyses and data management were performed in SAS version 9.4 (SAS Institute, Cary, NC, USA) and R version 4.2.1 (R Foundation for Statistical Computing, Vienna, Austria) [8].

### Post hoc validation analysis

The primary study population was tested in regard to the number of included patients and overall incidence in a validation cohort identified by use of a new set of inclusion criteria, previously validated by Sreih *et al.* in the setting of anti-neutrophil cytoplasmic antibody (ANCA)-associated vasculitis (AAV) [9]. The validation method was modified to fit anti-GBM disease as well as the Danish registries and registration routines (Supplementary Table S1). Finally, in the assessment of our primary study population, we examined the percentage of patients included from four of five administrative regions in Denmark (from where we had full serology sampling during 2015–2018) who either had positive anti-GBM serology, a confirmatory kidney biopsy or both, endorsing the ICD-10-based diagnosis.

### Ethics

Retrospective registry-based studies including anonymized data do not require ethical approval in Denmark. This study was approved by the Danish Data Protection agency (ref. GEH-2014-018, I-Suite no.: 02736 and VD-2018-292, I-Suite no.: 6536).

## RESULTS

### Population demographics: total population

**Figure 1:**
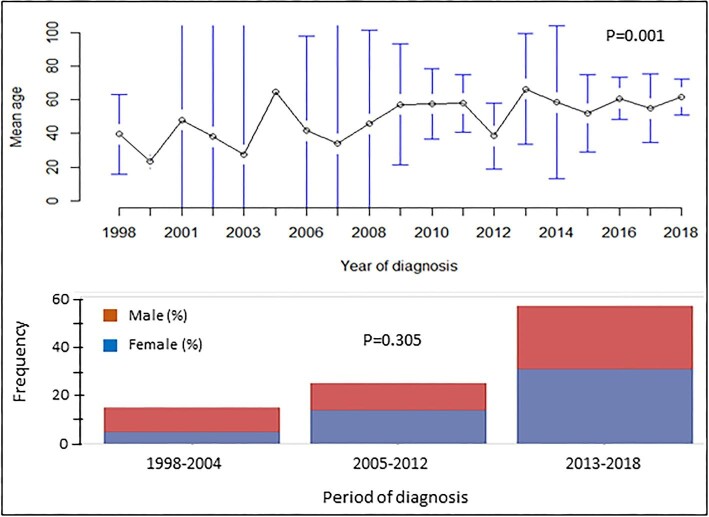
Changes in mean age at diagnosis and distribution of gender among patients with anti-GBM disease during 1998–2018. The difference in mean age was tested by the use of ANOVA.

Ninety-seven patients with incident anti-GBM disease were identified between 1998 and 2018. The median age at the time of diagnosis was 56 years [interquartile range (IQR) 46] with almost equal sex distribution (51.6% female) and a median follow-up of 6.1 years (IQR 9.2) (Table 1). The distribution of age and gender exhibited periodic effects with increasing age at diagnosis during the total study period (*P* for difference = .001) and a trend (*P* = .31) towards female predominance during the final 15 years of follow-up (Fig. 1). While there was a male predominance among patients <50 years of age, female gender was more common in patients >50 years of age (*P* = .02). Younger patients were more likely to have lung haemorrhage (*P* = .016), whereas older patients had a higher frequency of advanced disease judged by the length of their initial hospital stay (admission lasting >10 days or death within 10 days from admission) and more patients had a previous malignant diagnosis at baseline (Table 1).

**Table 1:** Baseline characteristics of patients with anti-GBM disease stratified on age.

Characteristics	Total	Age ≤50 years	Age >50 years	*P* for difference
Patients, *n*	97	38	59	–
Age (years), median (IQR)	56 (46)	–	–	–
Female, *n* (%)	50 (51)	14 (38.8)	36 (61.0)	.020
HTN, *n* (%)	43 (44.3)	13 (34.2)	30 (50.9)	.107
COPD, *n* (%)	7 (7.2)	≤3^a^	≤10^a^	.161
DM, *n* (%)	12 (12.4)	≤3^a^	≤10^a^	.088
CHF, *n* (%)	10 (10.3)	≤3^a^	≤10^a^	.190
IHD, *n*	≤3^a^	≤3^a^	≤3^a^	.158
Cancer, *n* (%)	14 (14.4)	≤3^a^	≤14^a^	.001

HTN: hypertension; COPD: chronic obstructive pulmonary disease; DM: diabetes mellitus; CHF: chronic heart failure; IHD: ischaemic heart disease.

^a^Exact number not filed due to the anonymization policy.

Thirty-four (35.1%) patients met the criteria for advanced disease severity, 36 (37.1%) had an intensive care unit (ICU) stay and 72 (74.2%) received PLEX. At the time of the last follow-up, 65 (67.0%) patients remained dependent on renal replacement therapy (dialysis or kidney transplantation), corresponding to a 5-year kidney survival of 35.2%. A total of 45 (58.4%), 60 (61.9%) and 61 (62.9%) patients had commenced haemodialysis at day 30, 180 and 360 after first day of admission, respectively (Table 2). None of the patients who started dialysis regained their kidney function beyond 60 days (the time used to define acute versus chronic dialysis). The 1-year kidney survival probability was 0.38 (SD 0.05), with a significant increase from 2010 onwards [1998–2004: 0.47 (SD 0.13), 2005–2011: 0.16 (SD 0.07), 2012–2018: 0.46 (SD 0.07); *P* = .035] (Supplementary Fig. S1). The number of patients with a prolonged hospital stay lasting >10 days remained constant over time; however, the percentage of patients diagnosed with hypertension at baseline increased. There was no temporal difference regarding diabetes, peripheral vascular disease, ischaemic heart disease, chronic obstructive pulmonary disease or congestive heart failure More detailed information on baseline characteristics stratified on year of inclusion (1998–2004, 2005–2011, 2012–2018) are shown in Supplementary Table S2.

**Table 2:** Outcome variables stratified by age.

Variable	Total	Age ≤50 years	Age >50 years	*P* for difference
RRT day 30	56 (58.0)	17 (44.7)	39 (66.1)	.104
RRT day 360	61 (62.9)	20 (52.6)	41 (69.5)	.132
Advanced disease	34 (35.1)	8 (21.1)	26 (44.1)	.020
PLEX	72 (74.2)	25 (65.8)	47 (79.7)	.127
ICU	36 (37.1)	17 (44.7)	19 (32.2)	.212
Lung haemorrhage	19 (19.6)	12 (31.6)	7 (11.9)	.017

**Figure 2:**
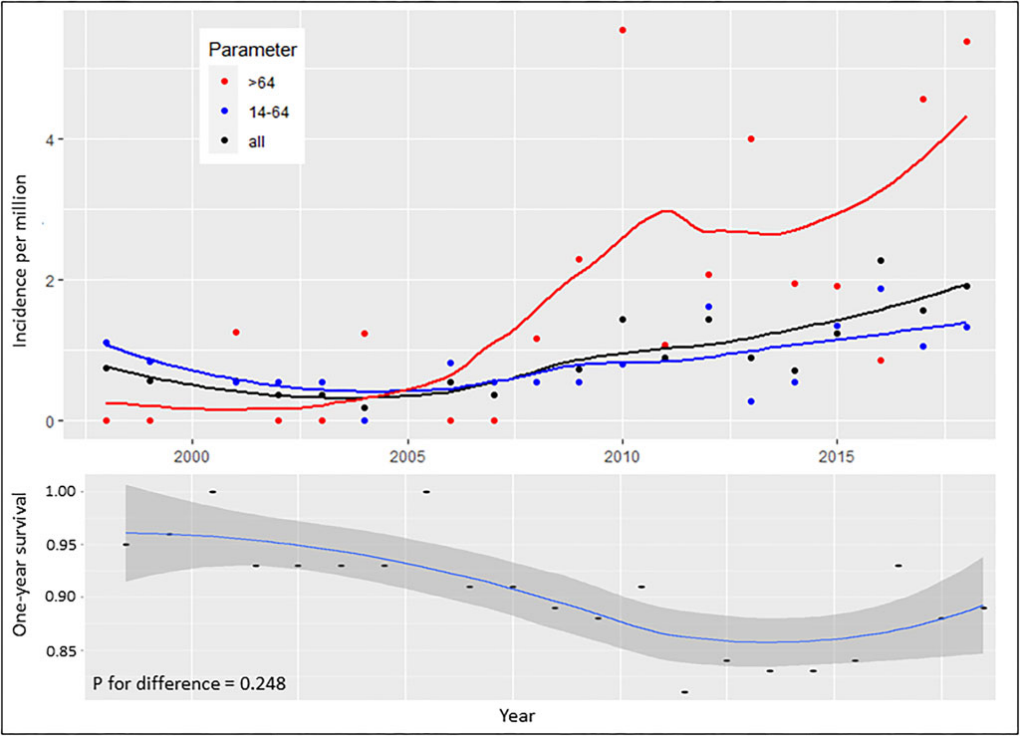
Age-stratified incidences of anti-GBM disease including 1-year survival probability in Denmark during 1998–2018.

**Figure 3:**
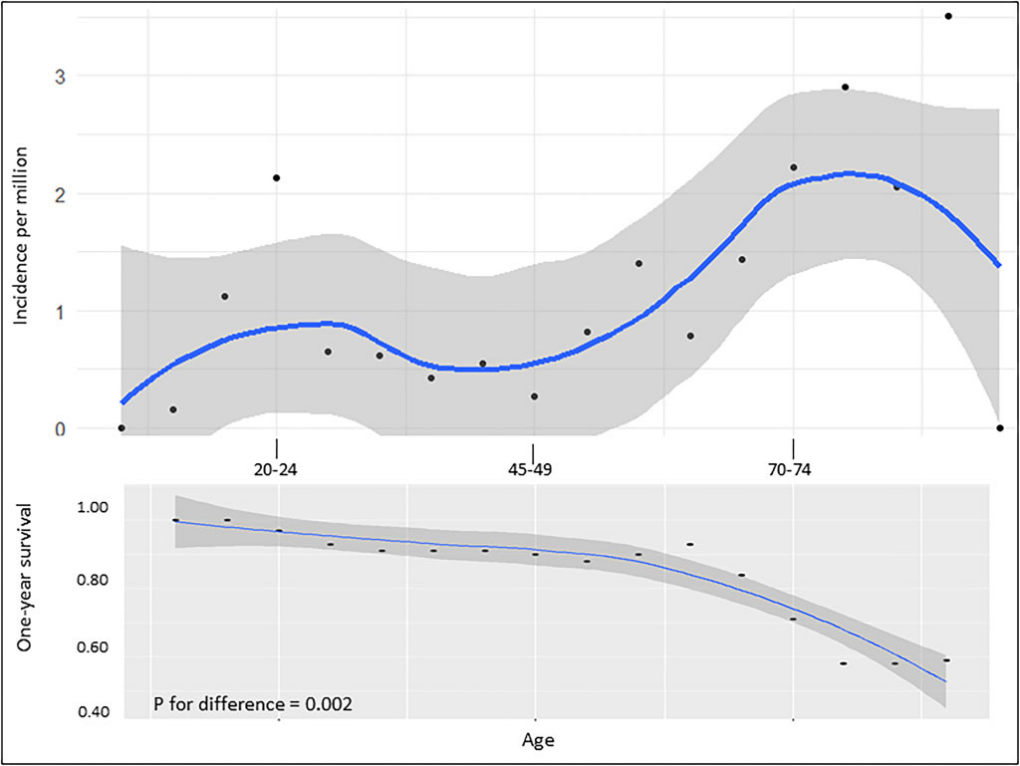
Age-specific incidences of anti-GBM disease including 1-year survival probability in Denmark during 1998–2018.

The overall incidence of anti-GBM disease during 1999–2018 was 0.91 cases/million/year (SD 0.6). Incidence increased with age [0.76 (SD 0.4), 1.5 (SD 1.04) and 4.9 (SD 2.6) cases/million/year for patients <45, 45–75 and >75 years, respectively (Fig. 2)] and over time [1998–2004: 0.50 (SD 0.2), 2005–2011: 0.80 (SD 0.4), 2012–2018: 1.4 (SD 0.5); *P* = .02 (Fig. 2)]. These findings were sustained in subanalyses of gender (Supplementary Fig. S2) and age groups >45 years, with the greatest increase in incidence among patients >64 years of age (Fig. 2). Moreover, age-stratified incidences for the total study period showed a bimodal distribution, peaking at 20–25 years and again at >70 years, and the highest incidences overall were registered among patients 85–90 years of age (Fig. 3). Cumulative frequencies of anti-GBM diagnoses exhibited significant seasonal variation, with the highest number of incident cases observed during spring and autumn (*P* for difference between spring and autumn versus winter and summer = .006; Supplementary Fig. S3).

### Survival analyses

**Figure 4:**
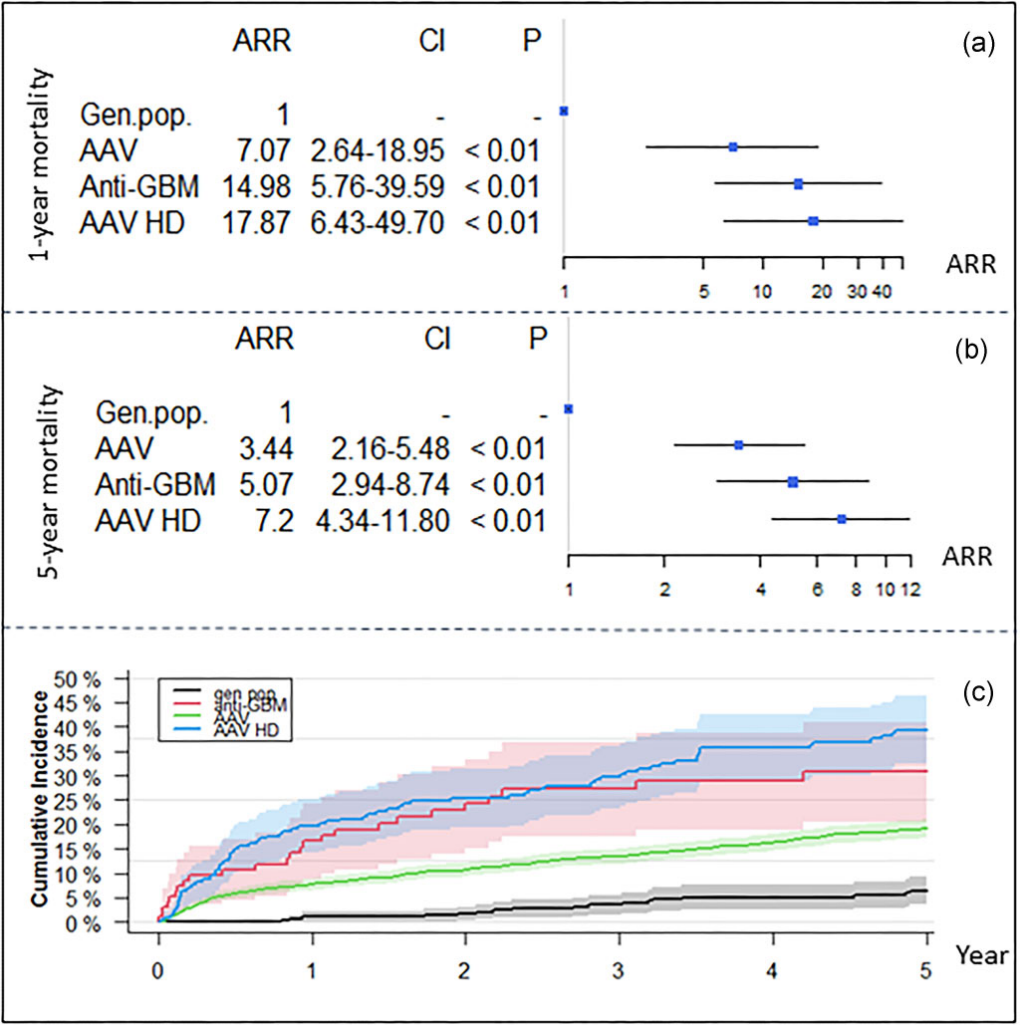
Risk of death in patients with anti-GBM disease as compared with an age- and sex-matched background population and patients with incident AAV with and without dialysis.

**Figure 5:**
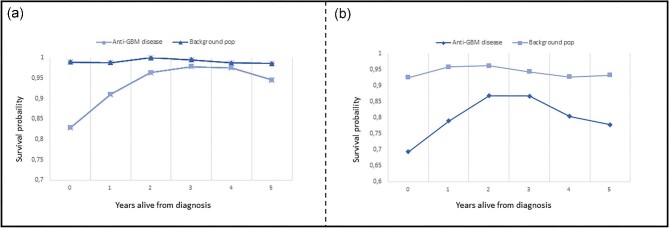
The 1-year (a) and 5-year (b) survival probability based on years alive from initial diagnosis as compared with an age- and sex-matched background population.

Thirty-five (35.5%) patients died, with a median time to death of 1.1 years (IQR 3.9) during the total study period, 26 of whom died during the first 5 years, corresponding to a 5-year mortality of 26.8%. The mean age at the time of death was 69.1 years (SD 17.6), with a numerical predominance of women (61%; *P* for difference = .17) and only 6.2% of the patients being <50 years old. The 1-year survival decreased with age (*P* for difference = .002; Fig. 3) but remained statistically unchanged over time (*P* for difference = .248; Fig. 2). The primary causes of death were cardiovascular disease (52.9%), vasculitis (23.5%), cerebral disease (23.5%) and infection (17.7%). The overall 1- and 5-year risk of death associated with anti-GBM disease was significantly increased compared with the matched background population [1-year: ARR 14.98 (CI 5.76–39.6), *P* < .001; 5-year: ARR 5.27 (CI 2.45–11.3), *P* < .001] and was comparable to that of patients with incident AAV dependent on dialysis at day 30 after first day of admission [1-year: ARR 0.82 (CI 0.48–1.41), *P* = .50]; 5-year: ARR 0.82 (CI 0.48–1.41), *P* = .50] (Fig. 4). One- and five-year survival probability, based on years alive from initial diagnosis relative to matched background populations exhibited increasing survival probabilities for patients who survived 1, 2 and 3 years after diagnosis, after which, probabilities started to decrease (Fig. 5, Supplementary Table S3).

### Population demographics: serology and double positivity

**Figure 6:**
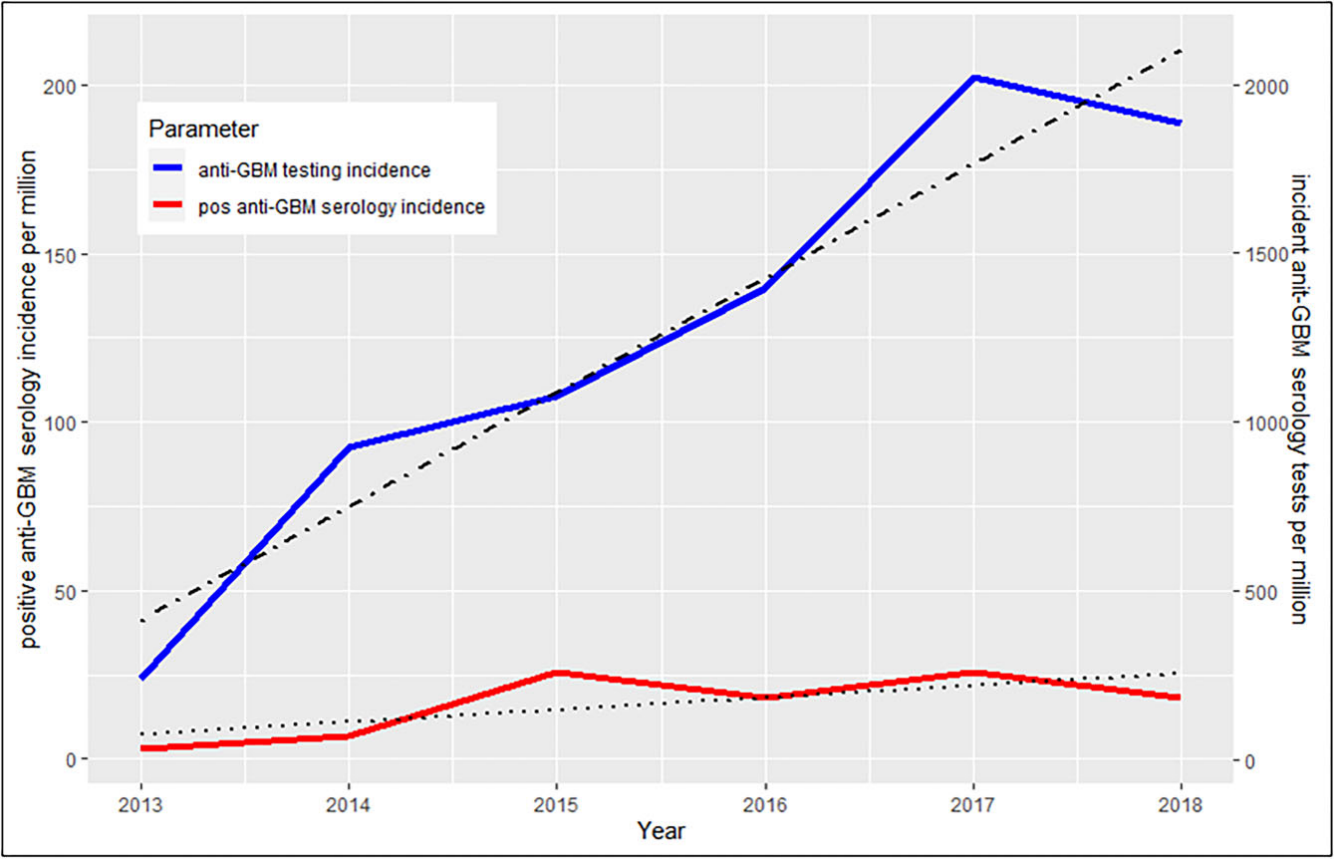
Incident anti-GBM serology testing and incident positive anti-GBM tests from four of five administrative regions in Denmark during 2013–2018.

Serological data were available from a subgroup of 32 ICD-10-confirmed patients with anti-GBM disease during 2013–2018. Anti-GBM serology testing obtained in this period increased more than positive anti-GBM tests (Fig. 6), and incident anti-GBM antibody positivity [16.3 cases/million/year (SD 9.5)] was higher than the corresponding ICD-10-based incidence [0.91 (SD 0.6)]. Of the 32 ICD-10-confirmed patients with serology, 40.6% showed double-positive serology with the coexistence of both anti-GBM antibodies and ANCA, and with a numerical preponderance of myeloperoxidase (MPO)-ANCA (53.8%). ‘Double-positive’ patients tended to be older, with a mean age of 62.3 years (SD 22.1) [compared with 56.1 years (SD 25.3) for single-positive GBM], they had a lower mean anti-GBM antibody concentration at the time of diagnosis [150.7 KU/l (SD 146.5) versus 333 KU/l (SD 1278.7), *P* = .04] and were more likely female (69.2%) (Supplementary Table S4).

### ICD-10 code validation and subanalyses

By applying two of the three validated inclusion criteria (ICD-10 codes and encounter type), 81 patients with anti-GBM disease were identified, corresponding to 83.5% of the original study population. By additionally including the specialty involved in their care, 80 patients were identified as having anti-GBM disease, corresponding to 82.5% of the original study population (all patients identified were contained in the original cohort). The overall anti-GBM disease incidence based on the three validated inclusion criteria was 0.79 cases/million/year (SD 0.6), with a significant increase over time [1998–2004: 0.41 (SD 0.2), 2005–2011: 0.54 (SD 0.3), 2012–2018: 1.28 (SD 0.6); *P* < .001]. Finally, 31 patients with ICD-10-confirmed anti-GBM with corresponding serological data were identified, of whom, 21 (67.7%) had confirmed positive anti-GBM serology in concentrations above the upper reference level, and 18 (58.1%) patients were registered with a confirmatory kidney biopsy showing anti-GBM disease. Overall, 26 (83.9%) patients had either positive serology, a confirmatory kidney biopsy or both.

In subanalyses of the 72 patients receiving PLEX as part of their initial treatment, overall mortality (34.7%), 5-year mortality (29.2%), median time to death [1.0 years (IQR 1.9)] and mean age at the time of death [54.4 years (SD 23.2)] were comparable to what was found in the total study population, as were the distribution of age, gender and other included baseline variables (Supplementary Table S1). Further details on differences in baseline characteristics between patients receiving PLEX and patients not receiving PLEX are shown in Supplementary Table S5. Subanalyses of patients dependent on dialysis within 30 days from the initial diagnosis showed a significantly increased 5-year mortality for patients with anti-GBM disease on dialysis as compared with anti-GBM patients not receiving dialyses, with an ARR of 2.36 (CI 1.03–5.40; *P* = .043) (Supplementary Fig. S4). Baseline characteristics for patients with anti-GBM disease stratified on dialysis at day 30 after diagnosis are shown in Supplementary Table S6.

## DISCUSSION

As in the current research, previous studies have suggested a bimodal relationship between age and anti-GBM incidence, as well as a possible seasonal relationship [10–12], however, to our knowledge, we are the first to demonstrate a time-dependent increase in incident anti-GBM disease during the previous 2 decades, as suggested in both the ICD-10- and serology-based analyses (Fig. 5). Nonetheless, similar observations have been noted in other autoimmune diseases, especially those where older age correlates with increasing incidence [13, 14], and may link to the demographic transition seen in many industrialized countries with low birth and death rates, resulting in a relative and actual increase in the older population over time. Additionally, as the increase in incident anti-GBM disease coincided with increased anti-GBM testing during 2013–2018, with a corresponding but more modest increase in positive anti-GBM tests over the same period, these findings may relate to greater disease awareness, especially in patients of older age, as well as better availability and access to tests, as has been previously demonstrated in the case of AAV in Denmark [3]. This notion is further substantiated by the augmented increase in incidence among patients >64 years of age, as the likelihood of identifying incident cases increases in a population where the a priori risk of the disease is high. Taken together, the observed increase in incidence likely relates to a combination of better disease awareness, greater testing frequency and a demographic shift towards a larger population of older people.

The bimodal distribution of age-stratified incidences observed in the current study was further linked to differences in phenotype. As such, we found a higher frequency of pulmonary haemorrhage among younger men and a higher frequency of ANCA and anti-GBM double positivity in older women, with a preponderance of MPO-ANCA. This finding is consistent with previous studies in AAV showing an increasing incidence in older women with MPO positivity [15]. This tendency could potentially influence the incidence in anti-GBM disease, as 40% of patients with an ICD-10-confirmed anti-GBM diagnosis were ANCA–anti-GBM double positive in the present study. Similar high rates of double positivity among patients with anti-GBM disease have been described in other studies [15–17]; however, conversely, only 5–10% of the patients initially diagnosed with AAV have been shown to exhibit ANCA and anti-GBM co-occurrence [2]. This relationship between ANCA and anti-GBM serology may pinpoint a potential causal association between AAV and anti-GBM disease, where existing AAV disease may expose parts of the glomerular or alveolar basement membrane, which in the right immunologic milieu will result in the formation of anti-GBM autoantibodies and subsequently anti-GBM disease, with a mixed phenotype of both diseases, as also suggested previously [18]. Incidentally, double-positive patients in the current study were also found to have lower concentrations of anti-GBM antibodies as compared with those with exclusively anti-GBM positivity. Hence the phenotypic difference between patients with disease onset early in life versus those who are diagnosed later in life may rely on differences in the pathogenesis that putatively relates to age.

There may be numerous reasons connecting autoimmunity to older age, such as genetic mutations and epigenetic alterations. In this regard, Beck *et al*. [19] recently identified a new disease entity called the VEXAS syndrome (vacuoles, E1 ubiquitin conjugating enzyme, X-linked, autoinflammation, somatic), which is caused by somatic mutations of the *UBA1* gene in haematopoietic progenitor cells of older men, inflicting a range of inflammatory and haematologic symptoms. The pathophysiology of the VEXAS syndrome, with an X-linked somatic mutation exclusively occurring in men >50 years of age being the culprit lesion in contrast to the usual heritable genetic mutations, highlights a new insight into how age can affect autoimmunity [20]. Moreover, epigenetic alterations of genes, that essentially control weather a specific gene can be expressed or not, has been linked to senescence [21]. In this regard, DNA methylation dysregulation in CD4^+^ T cells has been linked to autoimmunity in patients with systemic lupus erythematosus (SLE) [22, 23]. Also recently, a genetic risk score based on single-nucleotide polymorphisms (SNPs) in human leucocyte antigen (HLA) and non-HLA gene loci showed a linear relationship between genetic risk and age at SLE diagnosis, and interestingly, non-HLA SNPs were associated with older age at diagnosis [24], again linking age to an autoimmune genotype that putatively explains in part the observed differences in SLE phenotype when stratified on age and gender [25], and which may apply to other autoimmune diseases including anti-GBM disease.

Despite the increasing age at diagnosis, 1-year survival probability remained unchanged and 1-year renal survival probability increased during follow-up, which may indicate some degree of progress in our management of these patients, as one would expect increasing mortality and decreasing renal survival with increasing age. Also, considering that double positivity with lower peak anti-GBM concentrations appears more frequently among older anti-GBM patients, these individuals may have less kidney injury and subsequently better renal outcome. However, the main reason for the observed increase in renal survival is putatively explained by earlier disease detection due to the marked increase in anti-GBM testing incidence documented between 2013 and 2018, which also may explain the marked increase in incidence among patients >64 years of age, as discussed above. Nonetheless, overall mortality and the rate of ESKD, with 65% of the patients being dependent on RRT after 5 years, and a 5-year mortality of ≈25%, remained in concordance with previous studies [15, 26]. Also, as expected, 1- and 5-year risk of death was markedly increased with anti-GBM disease as compared with the background population, as well as AAV patients without renal involvement; however, it remained comparable to a cohort of dialysis-dependent patients with AAV, stressing the impact of renal involvement. Accordingly, 5-year mortality was significantly increased in patients with anti-GBM disease dependent on dialysis at day 30 after initial diagnosis as compared with anti-GBM patients who did not receive dialysis. Interestingly, cumulative incidences of death in patients with anti-GBM disease and RRT-dependent AAV patients seemed to diverge over time, with higher cumulative incidences in the latter group, which might indicate the relapsing nature of AAV as opposed to anti-GBM disease.

Collectively, these findings highlight two very central issues concerning kidney involvement in anti-GBM disease; namely, that renal involvement, with the need for RRT at diagnosis, is a marker of severe high-risk disease with a poor chance of renal survival [15, 17] and loss of kidney function is among the most imminent concerns in patients who survive the initial course of the disease, as ESKD inherently increases the risk of death. This is also suggested in the current study, where 1- and 5-year survival incrementally increased for patients alive 1, 2 and 3 years after initial diagnosis, relative to a matched background population, where it again started to decline, in agreement with the high frequency of ESKD in the cohort. As such, the need for improved treatment strategies and optimized methods for identifying patients at risk of contracting anti-GBM disease certainly remains.

In an attempt to identify new and more effective treatment strategies, a recently published phase 2 study proposed an innovative and potentially advantageous treatment approach based on *in vivo* cleavage of IgG by an IgG-degrading endopeptidase (imlifidase) derived from *Streptococcus pyogenes*, which rapidly cleaves all circulating IgG, including the anti-GBM disease-causing IgG directed against type IV collagen in the GBM. Although results are very preliminary, this treatment strategy as an add-on to the standard of care showed promising outcomes as compared with earlier publications [27]. Currently, a phase 3 study of imlifidase is being planned, which may potentially pave the way for a new aera of more effective anti-GBM disease management.

### Limitations

The results might have been affected by the limited sensitivity of the applied diagnostic codes. In this regard, we were unable to assess the sensitivity of the applied diagnostic codes, only the PPV. Also, PLEX and lung haemorrhage was putatively underestimated in the first part of the study period, due to insufficient coding before 2004, which partly explains why not all included patients based on ICD-10 codes were registered as receiving PLEX. Nonetheless, subanalyses excluding patients without PLEX as part of their initial treatment, hence reducing the risk of case admixture, supported the main results of the study. The percentage of patients with hypertension at baseline increased during the study period. This may pertain to altered coding habits and increasing age during the study; however, specifically it is related to altered treatment and diagnostic guidelines that were implemented during the same period [28, 29], as we included treatment with two or more antihypertensive medications as a proxy for hypertension. Also, the accuracy of hospital codes denoting ESKD have improved over time and such variation may have weakened the observed increase in renal survival over time.

## CONCLUSIONS

The incidence of anti-GBM disease increased over the test period, possibly related to better disease awareness, greater testing frequency and a demographic shift towards a larger population of older people. Mortality remained high and was comparable with an age- and sex-matched cohort of dialysis-dependent AAV patients. The diagnostic code identifying anti-GBM disease in the Danish registries (ICD-10 DM310A) is applicable for epidemiologic research.

## Supplementary Material

sfad261_Supplemental_File

## Data Availability

The data underlying this article will be shared upon reasonable request to the corresponding author.
