# Supplementary material for: Age- and time-dependent increases in incident anti-glomerular basement membrane disease: a nationwide cohort study
Source: Clin Kidney J. 2023 Oct 16;17(1):sfad261. doi: 10.1093/ckj/sfad261 (PMC10768786; doi:10.1093/ckj/sfad261)
Supplement: sfad261_Supplemental_File [file sfad261_supplemental_file.docx]

**Age and time-dependent increase in incident anti-glomerular basement membrane (anti-GBM) disease**

**-a nation-wide cohort study**

Supplementary material

Figure 1:

One-year renal survival with a significant change over time (P=0.035)


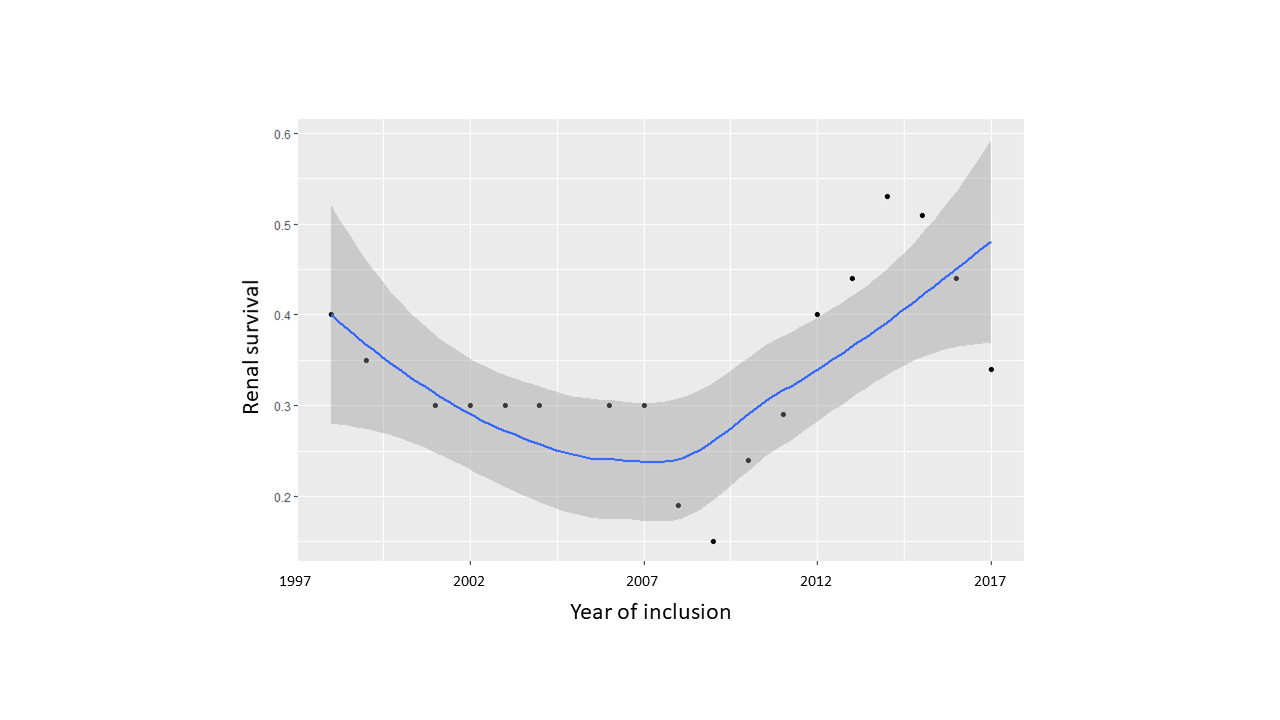


Figure 2:

Male and female incidence of anti-GBM disease during 1998-2018


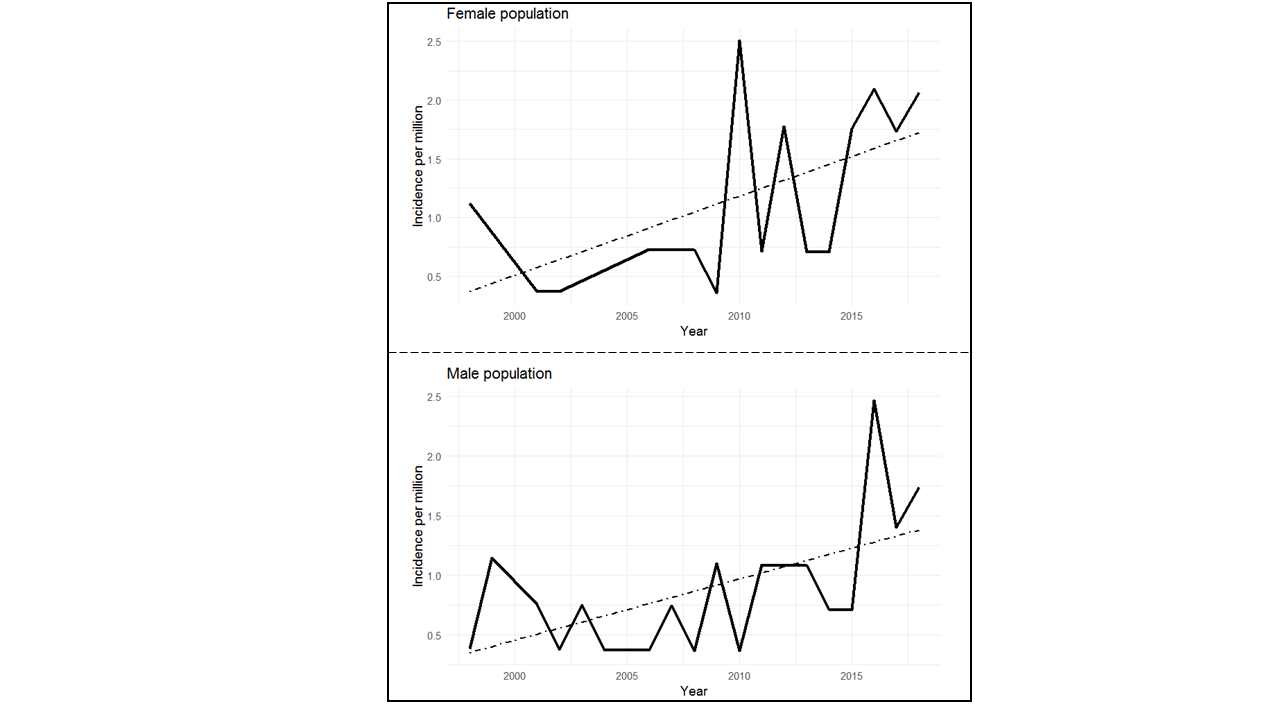


Figure 3:

Seasonal variation in cumulative frequencies of incident Anti-GBM diagnoses during 1998-2018


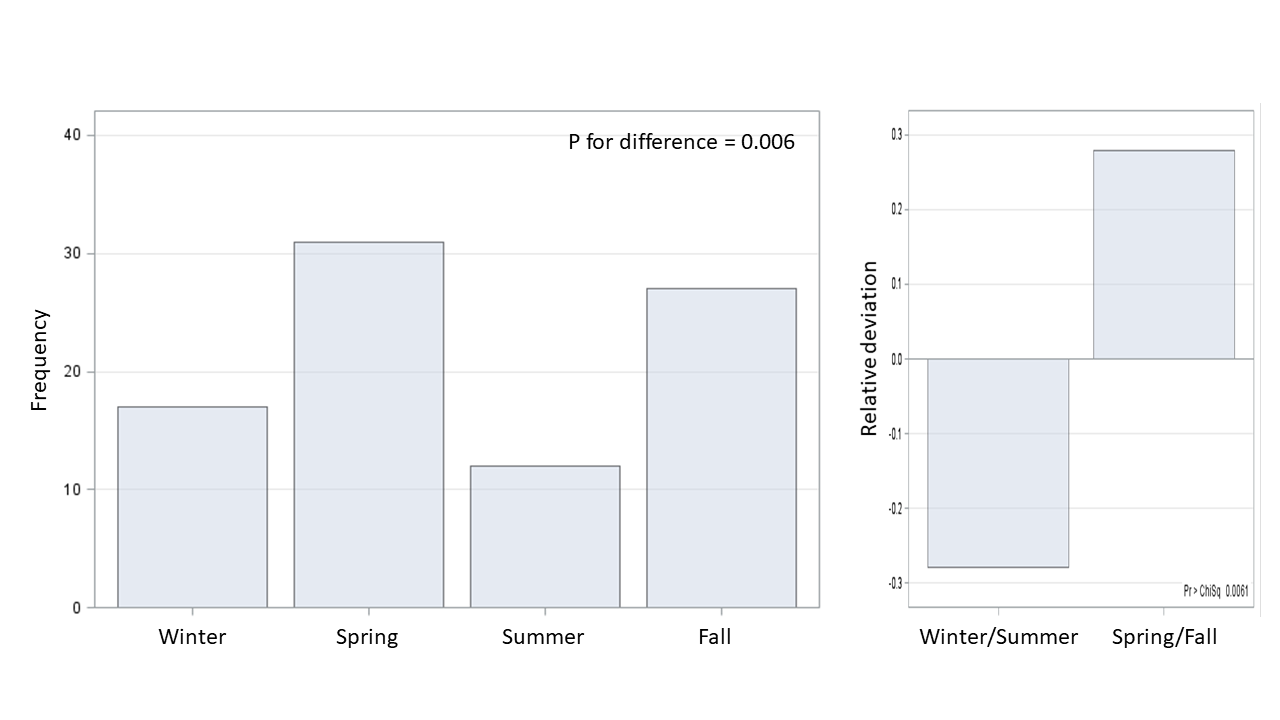


Figure 4:

Survival probability stratified on dialysis dependency at day 30 after

Initial diagnosis. (Black line: no dialysis. [P-value 0.012])


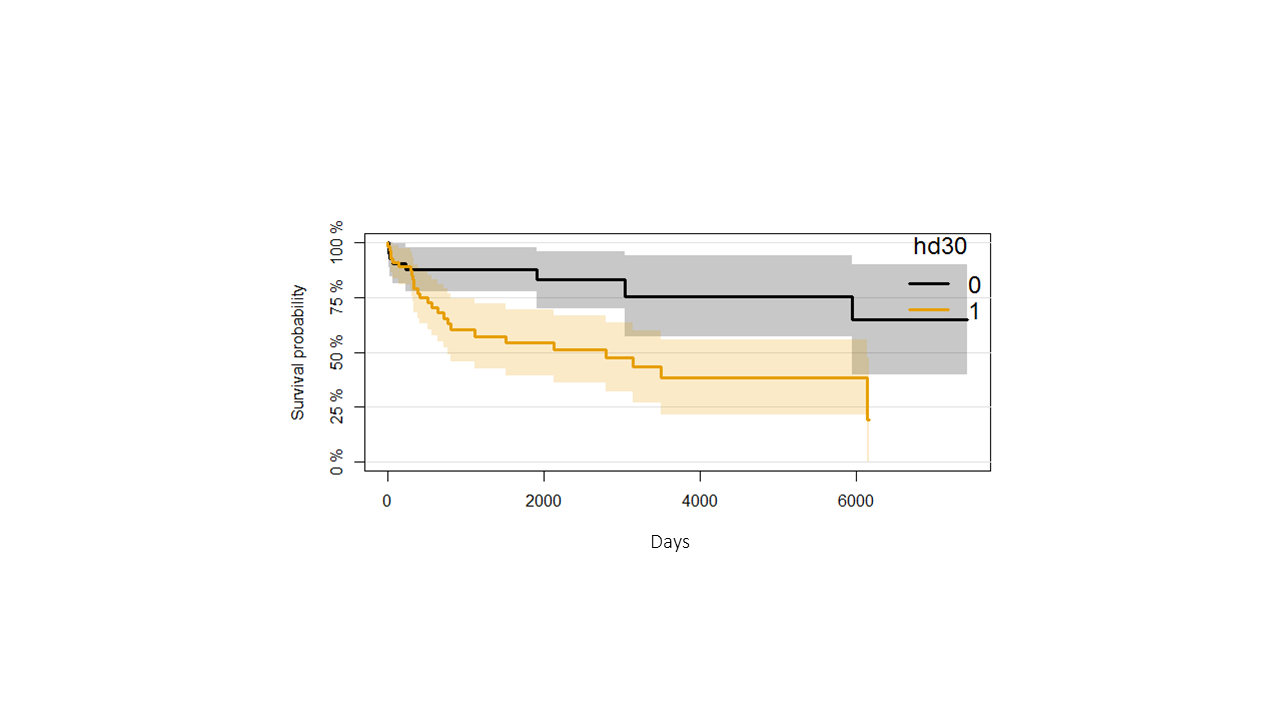


Table 1.

| Post hoc validation analysis |
| --- |
| The validation cohort was identified in the National Patient Registry using a combination of 1) ICD10 codes (ICD10: DM31.0A), 2) encounter type (either “inpatient” defined as an ICD10 code submitted on 3 consecutive days or ‘outpatient’ defined as two ICD9 codes submitted 3 months apart) and, 3) specialty involved in the care (internal medicine, otorhinolaryngology, and pediatric medicine). The latter criterion was slightly modified in the context of current study as some Danish hospitals did not differentiate between the specialties within internal medicine. Also, pediatric medicine was not included in the original criterion although there are occasionally cases of anti-GBM disease in patients younger than 18 years of age. Accordingly, we included internal medicine and pediatric medicine in in this criterion. |

Table 2.

| **Bassline characteristics of patients with Anti-GBM disease stratified on year of inclusion** | | | | |
| --- | --- | --- | --- | --- |
|  | 1998-2004 | 2005-2011 | 2012-2018 | P for difference |
| N | 15 | 25 | 57 | - |
| Female Sex, n (%) | 5 (33.3) | 14 (56.0) | 31 (54.4) | 0.305021 |
| Age, mean (SD) | 37.9 (19.2) | 52.4 (21) | 56.2 (23.2) | 0.007345 |
| Severe disease, n (%) | 4 (26.7) | 8 (32.0) | 22 (38.6) | 0.643990 |
| ICU, n (%) | 6 (40.0) | 15 (60.0) | 15 (26.3) | 0.014182 |
| PLEX, n (%) * | 6 (40.0) | 22 (88.0) | 44 (77.2) | 0.002572 |
| Lung hemorrhage, n (%) | 4 (26.7) | 7 (28.0) | 8 (14.0) | 0.257150 |
| HD, n (%) | 7 (46.7) | 18 (72.0) | 28 (49.1) | 0.127157 |
| IHD, n (%) | ≤3 | ≤3 | ≤3 | 0.345057 |
| DM, n (%) | <3 | <3 | 8 (14.0) | 0.310188 |
| HTN, n (%) | 7 (46.7) | 20 (80.0) | 45 (78.9) | 0.029377 |
| COPD, n (%) | ≤3 | ≤3 | ≤3 | 0.541221 |
| Cancer, n (%) | ≤3 | ≤3 | 11 (19.3) | 0.264104 |
| *PLEX was insufficiently registered before 2004  ICU: intensive care unit; PLEX: plasma exchange; HD: haemodialysis within 30 days;  IHD: ischemic heart disease; DM: Diabetes; HTN: hypertension.  COPD: chronic obstructive pulmonary disease | | | | |

Table 3

| **One- and five-year survival probability, based on years alive from initial diagnosis relative to matched background populations** | | | | | |
| --- | --- | --- | --- | --- | --- |
| 5-year survival probability | | | 1-year survival probability | | |
| Years alive | Anti-GBM | Backgr. Pop | Years alive | Backgr. pop | antiGBA |
| 0 | 0,693 | 0,925 | 0 | 0,989 | 0,828 |
| 1 | 0,79 | 0,958 | 1 | 0,988 | 0,91 |
| 2 | 0,868 | 0,962 | 2 | 1 | 0,963 |
| 3 | 0,867 | 0,943 | 3 | 0,995 | 0,978 |
| 4 | 0,804 | 0,927 | 4 | 0,987 | 0,975 |
| 5 | 0,778 | 0,932 | 5 | 0,986 | 0,945 |
|  | | | | | |

Table 4

| **Subgroup of patients with incident ICD10-confirmed anti-GBM disease with serology access between 2013-2018** | | | |
| --- | --- | --- | --- |
|  | Total | Anti-GBM | Double positive |
| N | 32 | 19 (59.4) | 13(40.6) |
| Age (SD) | 58.5 (23.9) | 56.1 (25.3) | 62.3 (22.1) |
| Female, % | 50% | 36.8% | 69,2% |
| Anti-GBM, KU/L (SD) | 195.5 (248.4) | 333 (278.7) | 150.7 (146.5) |
|  | | | |

Table 5

| **Bassline characteristics of patients with Anti-GBM disease stratified on plasmaexchange** | | | |
| --- | --- | --- | --- |
|  | PLEX | No PLEX | Total |
| N | 72 | 25 | 97 |
| Female Sex, n (%) | 38 (52.8) | 13 (52.0) | 50 (51) |
| Age, median (IQR) | 59.5 (49) | 45.0 (32.0) | 56 (46) |
| Severe disease, n (%) | 29 (40.3) | 5 (20.0) | 34 (35.1) |
| ICU, n (%) | 28 (38.9) | 8 (32.0) | 36 (37.1) |
| Lung hemorrhage, n (%) | 12 (16.7) | 7 (28.0) | 19 (19.6) |
| HD, n (%) | 52 (72.2) | 9 (36.0) | 61 (62,9) |
| IHD, n (%) | ≤3 | ≤3 | ≤3 |
| DM, n (%) | 7 (9.7) | 5 (20.0) | 12 (12.4) |
| HTN, n (%) | 33 (45.8) | 10 (40.0) | 43 (44.3) |
| COPD, n (%) | ≤3 | ≤3 | 7 (7.2) |
| Cancer, n (%) | 9 (12.5) | 5 (20.0) | 14 (14.4) |
| *PLEX was insufficiently registered before 2004  ICU: intensive care unit; PLEX: plasma exchange; HD: haemodialysis;  IHD: ischemic heart disease; DM: Diabetes; HTN: hypertension.  COPD: chronic obstructive pulmonary disease | | | |

| **Bassline characteristics of patients with Anti-GBM disease stratified on dialysis day 30 after initial diagnosis** | | | |
| --- | --- | --- | --- |
|  | Dialysis | No dialysis | Total |
| N | 56 | 41 | 97 |
| Female Sex, n (%) | 30 (53.6) | 20 (48.8) | 50 (51) |
| Age, median (IQR) | 67.5 (39.5) | 45.0 (39.0) | 56 (46) |
| Severe disease, n (%) | 21 (37.5) | 13 (31.7.0) | 34 (35.1) |
| ICU, n (%) | 24 (42.9) | 12 (29.3) | 36 (37.1) |
| Lung hemorrhage, n (%) | 10 (17.9) | 9 (22.0) | 19 (19.6) |
| PLEX* | 48 (85.7) | 24 (58.5) | 44 (77.2) |
| IHD, n (%) | ≤3 | ≤3 | ≤3 |
| DM, n (%) | ≤10 | ≤3 | 12 (12.4) |
| HTN, n (%) | 31 (55.4) | 12 (41.0) | 43 (44.3) |
| COPD, n (%) | ≤3 | ≤3 | 7 (7.2) |
| Cancer, n (%) | 8 (14.3) | 6 (14.6) | 14 (14.4) |
| *PLEX was insufficiently registered before 2004  ≤: exact number not filed due to the anonymization policy  ICU: intensive care unit; PLEX: plasma exchange; HD: haemodialysis;  IHD: ischemic heart disease; DM: Diabetes; HTN: hypertension.  COPD: chronic obstructive pulmonary disease | | | |
